# Alterations of peripheral cytokines, BDNF, and surface-based morphometry indices in T2DM patients without cognitive impairment

**DOI:** 10.3389/fnins.2023.1141261

**Published:** 2023-04-11

**Authors:** Wenjiao Lyu, Yuna Chen, Kui Zhao, Xin Tan, Ye Wu, Shijun Qiu

**Affiliations:** ^1^Department of Radiology, The First Affiliated Hospital of Guangzhou University of Chinese Medicine, Guangzhou, Guangdong, China; ^2^Department of Endocrinology, The First Affiliated Hospital of Guangzhou University of Chinese Medicine, Guangzhou, Guangdong, China; ^3^School of Computer Science and Engineering, Nanjing University of Science and Technology, Nanjing, Jiangsu, China

**Keywords:** type 2 diabetes mellitus, magnetic resonance imaging, cytokines, brain-derived neurotrophic factor, cognitive impairment, surface-based morphometry

## Abstract

**Purpose:**

This study aimed to investigate potential biological mechanisms underlying cognitive function alterations in Type 2 diabetes mellitus (T2DM) patients by integrating cortical morphology with peripheral cytokine levels and brain-derived neurotrophic factor (BDNF) levels, and to offer potential insights for the early detection of T2DM-related cognitive impairment.

**Methods:**

This study included 16 T2DM patients with a Montreal Cognitive Assessment (MoCA) score of at least 26 points, as well as 16 healthy controls with normal cognitive function. The participants also completed the digit span test and digit symbol substitution test. Participants’ serum levels of Interleukin 4 (IL-4), IL-6, IL-10, tumor necrosis factor-alpha (TNF-α), interferon-gamma (IFN-γ), and BDNF were also examined. Each subject underwent a high-resolution 3T structural brain MRI scan. Based on the aparc. a2009s atlas, we calculated the cortical thickness, sulcus depth, gyrification index, and fractal dimension for each participant using surface-based morphometry (SBM). Correlation analysis between cognitive measures, serum levels of cytokines and BDNF, and SBM indices were further performed.

**Results:**

The levels of IL-4 and BDNF showed significant group differences. In the T2DM group, the sulcus depth exhibited a significant decrease in the left transverse frontopolar gyri and sulci, as well as in the right pole-occipital; the fractal dimension showed a significant increase in the right posterior-dorsal part of the cingulate gyrus; and the gyrification index significantly increased in the left inferior part of the precentral sulcus and right triangular part of the inferior frontal gyrus. Correlation analysis revealed a significant positive correlation between IL-10 levels and the sulcus depth of left transverse frontopolar gyri and sulci; a significant positive correlation between the sulcus depth of the right pole-occipital and the digit span test-forward scores, and a significant negative correlation between the gyrification index of the left inferior part of the precentral sulcus and the digit span test-backward scores among T2DM participants.

**Conclusion:**

T2DM patients without cognitive impairment displayed reductions in IL 4 and BDNF levels, as well as significant alterations in their SBM indices, indicating that prior to the emergence of cognitive impairment, the SBM indices, peripheral cytokines, and BDNF may have altered in T2DM patients. IL-10 may lessen inflammation-related brain edema and preserve sulcus depth in T2DM patients through its anti-inflammatory activity.

## 1. Introduction

Type 2 diabetes mellitus (T2DM) is a disease caused by insufficient or relatively insufficient insulin secretion, with elevated blood glucose as the main manifestation. Currently, there are nearly 500 million people with T2DM worldwide, and the global incidence of T2DM is increasing year by year, which makes T2DM one of the diseases that threaten human health worldwide (Chatterjee et al., 2017; Sun et al., 2022). Among the many complications of T2DM, cognitive impairment has attracted increasing attention in recent years. Notably, individuals with T2DM have a 1.5 times increased risk for Alzheimer’s disease and other dementias (Biessels et al., 2006). Cognitive impairment can impair T2DM patients’ self-management, resulting in poor glycemic control or recurrent bouts of hypoglycemia, which can lead to cardiovascular events or death (Feil et al., 2012; Punthakee et al., 2012). Therefore, early diagnosis and early intervention of cognitive impairment in T2DM are critical to improving patient prognosis.

The development and application of magnetic resonance imaging (MRI) technology enabled the detection of structural alterations in the brain without invasion or radiation. A growing number of studies have been carried out using MRI to investigate the relationship between cognitive impairment and structural alterations in the brain in T2DM. Surface-based morphometry (SBM) is a cortical analysis approach that has recently gained popularity among researchers due to its ability to calculate various cerebral cortex indices and hence yield more cortical information than voxel-based morphometry (VBM) (Ahn et al., 2011). SBM has been applied to investigate cerebral cortical abnormalities in neuropsychiatric disorders, such as Alzheimer’s disease (Nho et al., 2012), schizophrenia (Palaniyappan and Liddle, 2012), autism (Nordahl et al., 2007), etc. Several studies have recently begun to explore alterations in SBM indices in T2DM patients (Kang et al., 2022; Shao et al., 2022). In addition, it has also been demonstrated that cytokines and brain-derived neurotrophic factor (BDNF) levels may be associated with the development of T2DM-related cognitive impairment (Zhen et al., 2013; Simo et al., 2017; Sun et al., 2018). Despite recent achievements in MRI analysis and molecular biology techniques, the underlying mechanisms of T2DM related cognitive impairment are far from well clarified. Moreover, to the best of our knowledge, there is still lacking the combination of SBM indices and cytokines levels to assess cognitive impairment in T2DM of the currently available studies. We speculate that combining these approaches to evaluate alterations in cortical structure as well as peripheral blood cytokines and BDNF in T2DM patients who have not yet exhibited cognitive impairment may give insights into the underlying mechanisms and early detection of T2DM-related cognitive impairment.

The purpose of this study was to investigate potential biological mechanisms underlying cognitive function alterations in T2DM patients by integrating cortical morphology with peripheral cytokine levels and BDNF levels, and to offer potential insights for the early detection of T2DM-related cognitive impairment.

## 2. Materials and methods

### 2.1. Participants

Patients with T2DM needed to meet the diagnostic criteria of the American Diabetes Association (ADA), have been diagnosed for more than 6 months, and had no previous diabetic crisis or diabetic complications. All subjects should be right-handed, have a MoCA score of at least 26, have at least 6 years of education, and have no history of cardiovascular disease, tumor, autoimmune system diseases, neurological diseases, psychiatric diseases, etc. In addition, none of the subjects have experienced trauma, surgery, infection, tobacco, alcohol, or drug use (except for regular use of blood glucose control drugs in T2DM patients) within 4 weeks before inclusion, and female subjects should not be pregnant or lactating. After strict inclusion and exclusion criteria, a total of 16 T2DM patients without cognitive impairment and 16 healthy controls were enrolled (ages between 29 and 65; recruitment period: June 2020 to June 2021). Detailed demographic information can be found in Table 1.

**TABLE 1:** Demographics and clinical characteristics of the participants.

	T2DM (*N* = 16)	HC (*N* = 16)	Statistics *(t*/*U*)	*P*-value
Gender (F/M)	5/11	8/8		0.473
Age (years)	45.625 ± 9.344	42.125 ± 10.436	0.999	0.326
Education (years)	12.0 (9.0, 14.0)	12.0 (10.5, 16.0)	100.00	0.287
Systolic BP (mmHg)	131.19 ± 18.16	126.94 ± 16.69	0.689	0.496
Diastolic BP (mmHg)	83.86 ± 8.73	81.94 ± 8.31	0.643	0.525
BMI	24.32 ± 2.56	23.19 ± 2.77	1.196	0.241
MoCA	27.00 (26.00, 28.25)	29.00 (27.75, 30.00)	76.50	0.050
DST_F	8.50 (7.00, 9.00)	8.00 (8.00, 9.00)	117.50	0.697
DST_B	4.50 (4.00, 5.00)	5.00 (4.00, 6.00)	104.00	0.360
DSST	50.19 ± 10.60	51.56 ± 14.20	–0.310	0.758
IL-4 (pg/mL)	5.68 (5.01,5.68)	5.68 (5.68,10.34)	76.00	0.031*
IL-6 (pg/mL)	1.61 (1.42, 2.07)	1.42 (1.32, 1.67)	161.50	0.207
IL-10 (pg/mL)	1.29 ± 0.51	1.274 ± 0.87	0.072	0.943
TNF-α (pg/mL)	2.54 ± 0.48	2.49 ± 0.42	0.309	0.760
IFN-γ (pg/mL)	9.76 (9.76, 10.09)	9.76 (9.76,11.06)	110.00	0.473
BDNF (pg/mL)	3026.69 ± 2031.81	5012.69 ± 2180.96	–2.664	0.012*
HbA1c (%)	8.13 ± 1.56	NA	NA	NA
FBG (mmol/L)	8.26 (7.58, 9.07)	NA	NA	NA

Data are presented as *N*, median (Q1, Q3), and mean ± SD. T2DM, type 2 diabetes mellitus group; HC, healthy control group; F, female; M, male; Systolic BP, systolic blood pressure; Diastolic BP, diastolic blood pressure; BMI, body mass index; MoCA, Montreal cognitive assessment; DST_F, digit span test forward; DST_B, digit span test backward; DSST, digit symbol substitution test; IL-4, interleukin 4; IL-6, interleukin 6; IL-10, interleukin 10; TNF-α, tumor necrosis factor-alpha; IFN-γ, interferon-gamma; BDNF, brain-derived neurotrophic factor; HbA1c, Hemoglobin A1c; FBG, fasting blood glucose. Fisher’s exact test was used for the statistical difference of gender. Two sample t-test was used for statistical group differences of age, systolic blood pressure, diastolic blood pressure, BMI, DSST, IL-10, TNF-α and BDNF. Non-parametric Mann–Whitney U test was performed for group comparison of the remaining variables. One asterisk (*) indicates the significant level with *P* < 0.05.

### 2.2. Cognitive tests

Prior to the acquisition of MRI images, each participant underwent a series of cognitive function tests. Chinese version of the Montréal Cognitive Assessment Scale-B (MoCA-B) (Nasreddine et al., 2005) test was primarily used to determine whether the subject with cognitive impairment, and only the subjects with MoCA scores ≥ 26 can be included in this study. Moreover, the digit span test (DST, including forward and backward versions) (Leung et al., 2011) and digit symbol substitution test (DSST) (Jaeger, 2018) were also used for a more comprehensive understanding of cognitive function.

### 2.3. Clinical measurements and laboratory examinations

Each subject was measured for blood pressure, height, and weight in addition to the usual clinical physical examination. Blood pressure was used to exclude patients with moderate to severe hypertension, and height and weight were used to calculate body mass index (BMI) and to exclude obese patients. All subjects will also have venous blood collected to measure serum levels of BDNF, interleukin 4 (IL-4), interleukin 6 (IL-6), interleukin 10 (IL-10), tumor necrosis factor-alpha (TNF-α), interferon-gamma (IFN-γ) and, in addition, hemoglobin A1c (HbA1c) and fasting blood glucose (FBG) in patients with T2DM to rule out hyperglycemic crisis and to assess the diabetic condition. Clinical examination and blood collection should be done in the morning on the day of the MRI test on an empty stomach.

### 2.4. Imaging data acquisition

A 3.0 Tesla MAGNETOM Prisma MRI scanner (Siemens Healthcare, Erlangen, Germany) equipped with a 64-channel head-neck coil was used to collect the MRI data. The parameters of 3D T1WI sequence as following: Field of View (FOV) = 256 mm^2^ × 256 mm^2^, slice thickness = 1.0 mm, number of slices = 192, voxel size = 1.0 mm^3^ × 1.0 mm^3^ × 1.0 mm^3^, Repetition time (TR) = 2530 ms, Echo time (TE) = 2.98 ms, integrated Parallel Acquisition Techniques (iPAT) = 2, flip angle = 7^°^, Echo spacing = 7.1 ms, Total acquisition time (TA) = 5 min 58 s.

### 2.5. Data preprocessing and SBM indices computation

Both T1-weighted imaging data preprocessing and SBM indices computation were carried out using SPM 12^1^ and computational anatomy toolbox (CAT12) software^2^ based on Matlab 2021b (The Mathworks Inc., Natick, MA, United States). Before data preprocessing, all the images were reviewed by two radiologists to confirm that there were no organic lesions in the brain, such as hemorrhages, infarcts, malformations, etc.

The image pre-processing steps were as follows: First, convert T1 imaging data from DICOM into NIfTI, then choose ICBM space template as Affine Regularisation and medium strength of SPM inhomogeneity correction for initial SPM 12 preprocessing, and then use the center of the mass algorithm to set origin, APRG approach for Skull- Stripping and optimized shooting method for spatial registration at CAT 12 preprocessing.

The SBM indices calculating steps were as follows: First, extract surface parameters, which contain sulcus depth, gyrification index, and fractal dimension in addition to cortical thickness. Then resample and smooth surface data with a 15 mm full-width half max (FWHM) Gaussian kernel for cortical thickness, and 20 mm FWHM Gaussian kernel for the other indices. Then extract surface values based on the aparc_2009 atlas (Destrieux et al., 2010).

### 2.6. Statistical analysis

**FIGURE 1:**
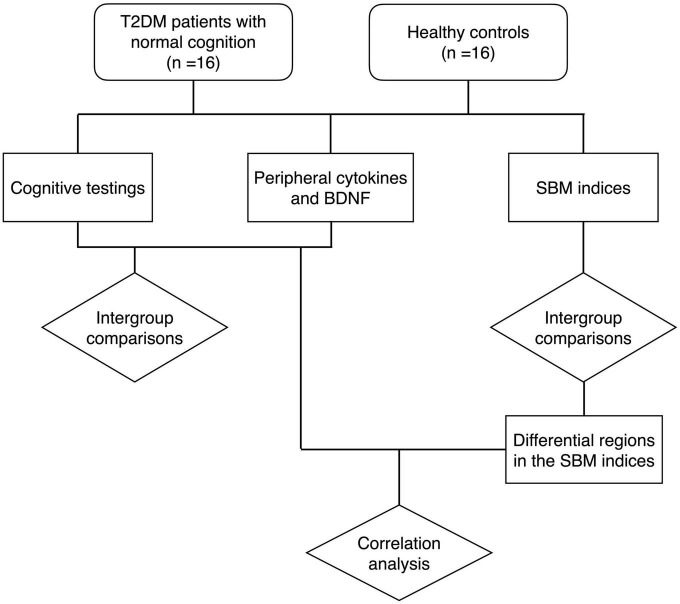
The flow chart of the study. T2DM, type 2 diabetes mellitus group; BDNF, brain-derived neurotrophic factor; SBM, surface-based morphometry.

Two-sample *t*-test, Mann-Whitney *U*-test and Fisher’s exact test were employed to assess differences between groups for demographic information, clinical data, and laboratory data, depending on the type of data and whether it corresponded to a normal distribution. For the detection of inter-group differences in SBM indices, gender was added as a covariate, and SPM software was used to perform factorial design, followed by two-sample *t*-tests with Holm-Bonferroni correction. The relationships between cognitive function scores, cytokines and SBM indices were calculated using Spearman partial correlation analysis controlled with gender. *P* < 0.05 was regarded as statistically significant. Statistical analyses were carried out using JASP software^3^ and R-Studio software.^4^ The flow of the study is shown in Figure 1.

## 3. Results

### 3.1. Demographics, clinical characteristics, and laboratory results

There were no significant differences in terms of gender, age, years of education, blood pressure and BMI between the two groups. Regarding the cognitive function scores, we only recruited T2DM patients without cognitive impairment. However, their MoCA scores were lower than those of healthy controls, although the difference was not statistically significant (*P* = 0.05). There were no significant group differences in the scores of DST and DSST between the two groups. Laboratory results of peripheral blood showed a significant decrease in the levels of IL-4 and BDNF in the T2DM group (*P* < 0.05). Other cytokines revealed no statistically significant differences between groups. Detailed information for the T2DM and HC groups is presented in Table 1.

### 3.2. Differential brain regions in the SBM indices between groups

**FIGURE 2:**
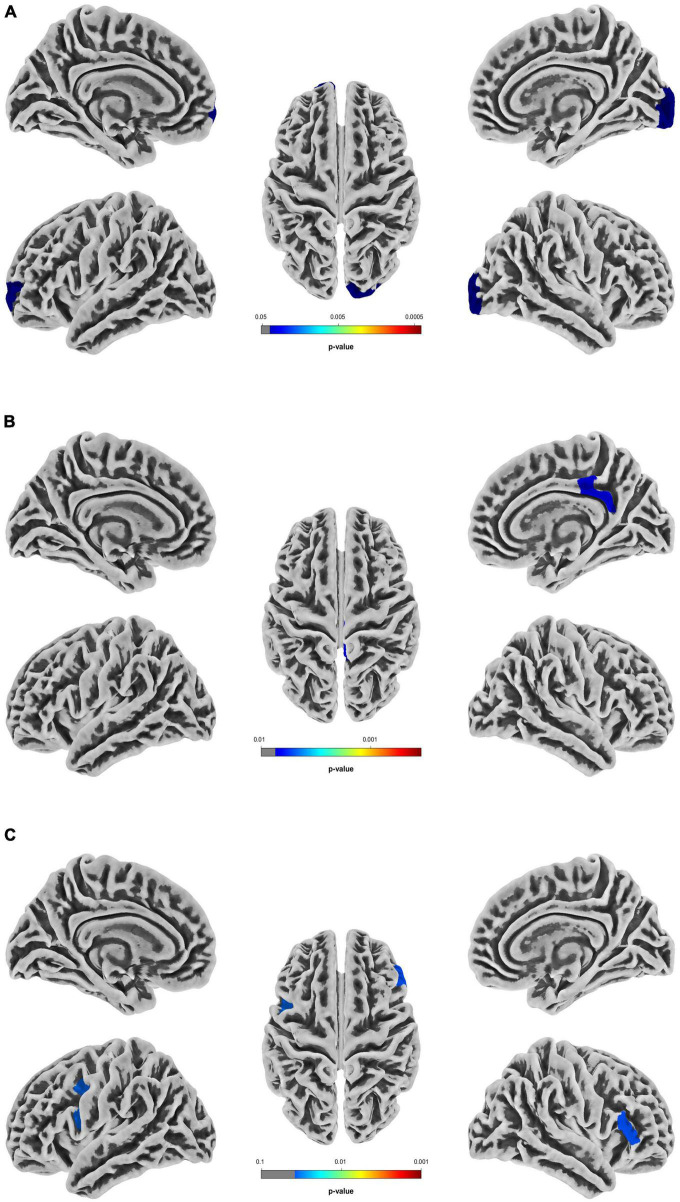
Differential brain regions in the SBM indices between T2DM patients with normal cognitive function and healthy controls. **(A)** Differential brain regions with intergroup differences in sulcus depth. **(B)** Differential brain regions with intergroup differences in fractal dimension. **(C)** Differential brain regions with intergroup differences in gyrification index. Two sample t-test, Holm-Bonferroni corrected. T2DM, type 2 diabetes mellitus group; SBM, surface-based morphometry.

In the T2DM group, the sulcus depth exhibited a significant decrease in the left transverse frontopolar gyri and sulci, as well as in the right pole-occipital (*P* = 0.040 and 0.017, respectively, Holm-Bonferroni corrected); the fractal dimension showed a significant increase in the right posterior-dorsal part of the cingulate gyrus (*P* = 0.007, Holm-Bonferroni corrected); and the gyrification index indicated a significant increase in the left inferior part of the precentral sulcus and right triangular part of the inferior frontal gyrus (*P* = 0.034 and 0.038, respectively, Holm-Bonferroni corrected). After adjustment for multiple comparisons, there was no significant difference in cortical thickness between groups. The information of the differential brain regions in the SBM indices is shown in Table 2, and the locations of the differential brain regions are shown in Figure 2.

**TABLE 2:** Information of the differential brain regions in the SBM indices between T2DM patients with normal cognitive function and healthy controls.

SBM index	Hemisphere	Atlas regions	*t*-value	*P*-value
Sulcus depth	Left	Transverse frontopolar gyri and sulci	–2.755	0.040*
Sulcus depth	Right	Pole occipital	–2.527	0.017*
Fractal dimension	Right	Posterior dorsal part of the cingulate gyrus	3.161	0.007**
Gyrification index	Left	Inferior part of the precentral sulcus	3.808	0.034*
Gyrification index	Right	Triangular part of the inferior frontal gyrus	2.353	0.038*

Two sample t-test, Holm-Bonferroni corrected. T2DM, type 2 diabetes mellitus group; SBM, surface-based morphometry. One asterisk (*) indicates the significant level with *P* < 0.05.

Two asterisks (**) indicates the significant level with *P* < 0.01.

### 3.3. Correlations between cognitive function scores, cytokines and SBM indices in the T2DM group

**FIGURE 3:**
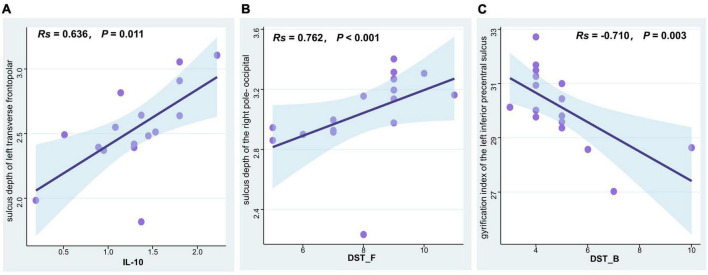
Correlation analysis of SBM indices, cytokine levels, and cognitive function scores in T2DM patients with normal cognitive function. **(A)** IL-10 levels were positively associated with the sulcus depth of left transverse frontopolar gyri and sulci (Spearman correlation, *R* = 0.636, *P* = 0.011). **(B)** The digit span test-forward scores were positively associated with the sulcus depth of the right pole-occipital (Spearman correlation, *R* = 0.762, *P* < 0.001). **(C)** The digit span test-backward scores were negatively associated with the gyrification index of the left inferior part of the precentral sulcus (Spearman correlation, *R* = –0.710, *P* = 0.003). T2DM, type 2 diabetes mellitus group; SBM, surface-based morphometry; IL-10, interleukin 10; DST_F, digit span test-forward; DST_B, digit span test-backward.

Spearman’s partial correlation analysis revealed a significant positive correlation between IL-10 levels and the sulcus depth of left transverse frontopolar gyri and sulci (*R* = 0.636, *P* = 0.011), a significant positive correlation between the sulcus depth of the right pole- occipital and the digit span test-forward (*R* = 0.762, *P* < 0.001), and a significant negative correlation between the gyrification index of the left inferior part of the precentral sulcus and the digit span test-backward (*R* = –0.710, *P* = 0.003) among T2DM participants (Figure 3).

## 4. Discussion

In this study, we examined the levels of peripheral blood cytokines and BDNF in T2DM patients without cognitive impairment, as well as healthy controls, and calculated indices such as sulcal gyrus depth, fractal dimension, gyrification index and thickness of the cerebral cortex utilizing the SBM approach. We noticed that T2DM patients had significant alterations in their cytokine levels, BDNF levels and SBM indices prior to the development of cognitive impairment. Further correlation analysis in T2DM patients revealed a non-negligible association between cytokine levels, SBM indices, and cognitive function scores. It is suggested that combining biological indices such as cytokines and BDNF with magnetic resonance imaging analysis technology can provide new insights into understanding the underlying mechanism of T2DM-related cognitive impairment and further explore biomarkers for its early diagnosis.

The cognitive decline induced by T2DM is an insidious process with complex causes, and the specific mechanism has not been clearly explained. Before mild cognitive impairment (MCI), T2DM patients may experience subtle cognitive changes in cognitive function, which is dubbed diabetes-associated cognitive decrements (Biessels and Despa, 2018). Despite the fact that we only recruited T2DM patients who were cognitively normal (none of them had a MoCA score below 26), it’s possible that some of them were undergoing a decline in cognitive function. Hence this period is crucial for unraveling the pathological mechanisms of T2DM-related cognitive impairment and developing therapeutic strategies in advance of effective intervention.

Many neurodegenerative diseases, including Alzheimer’s disease (AD), Parkinson’s disease, and mild cognitive impairment (Nagatsu et al., 2000; Guerreiro et al., 2007; Brosseron et al., 2014), are thought to be related to chronic neuroinflammation. Therefore, cytokines, as small molecular proteins that play an important role in the regulation of inflammation and immune system, have been focused on research (Dinarello, 2007). Elevated levels of TNF-α and IFN-γ have been found both in pathological brain specimens of postmortem patients with AD and in relevant animal model studies, so these two cytokines are considered to be neurotoxic (Brosseron et al., 2014; Uddin et al., 2022). Although conflicting results as to its association with cognitive function, IL-6 is generally thought to be related to either acute or chronic inflammatory pathophysiology of cognitive impairment (Brosseron et al., 2014; Lyra e Silva et al., 2021). One research found that T2DM patients with cognitive impairment had considerably greater peripheral blood IL-6 levels than T2DM patients without cognitive impairment (Anita et al., 2022), whereas another reported that T2DM patients’ IL-6 levels were significantly lower than those of healthy people (Yang et al., 2020). However, we did not observe any appreciable inter-group variations in TNF-α,IFN-γ, or IL-6. Our modest sample size, which will be enlarged for more analysis in the future, could be responsible for this. IL-4 and IL-10 are both anti-inflammatory cytokines that are commonly related to neuroprotection. It has been demonstrated that improved cognitive function is related to higher serum IL-4 and IL-10 concentrations in diabetic rats (Wang et al., 2020). IL-10 levels have been demonstrated to be considerably lower in T2DM patients than in healthy participants (Bashir et al., 2022), while elevated IL-10 levels have also been identified in studies related to nerve fiber injury (Magrinelli et al., 2015). BDNF is assumed to facilitate learning and long-term memory for its supporting neuronal growth and survival, boosting dendritic branching, and regulating synapses (Gadani et al., 2012; Lu et al., 2014). Lower serum BDNF levels have been linked to the severity of cognitive impairment (Shimada et al., 2014; Siuda et al., 2017), and numerous investigations have revealed that individuals with T2DM have considerably lower BDNF levels than healthy subjects (Zhen et al., 2013, 2018; Sun et al., 2018; Anita et al., 2022). We also find the levels of BDNF in T2DM group were significantly decreased, which is consistent with the conclusions of previous studies (Krabbe et al., 2007; Zhen et al., 2013). Animal studies have shown that IL-4 is associated with learning ability, memory formation, and cognitive function (Derecki et al., 2010; Gadani et al., 2012; Kipnis et al., 2012). Moreover, one research concluded that IL-4 can stimulate microglia to generate BDNF (McCormick and Heller, 2015), implying that IL-4 may directly act as a cytoprotective cytokine of neurons. There are, however, limited clinical investigations on the relationship between IL-4 and alterations in cognitive function in T2DM patients. According to our research, serum IL-4 levels in T2DM patients are considerably lower than in healthy participants. In conjunction with the BDNF changes mentioned above, our findings indicate that IL-4 and BDNF may be involved in the process of cognitive function alterations in T2DM patients. Although there were no significant differences in IL-10 levels across the groups, we did observe that in T2DM patients, serum IL-10 levels were positively linked with the sulcus depth of left transverse frontopolar gyri and sulci. Additionally, there was a substantial positive correlation between DST and the sulcus depth of the right pole-occipital. Therefore, we hypothesize that IL-10 is engaged in the anti-inflammatory process of T2DM patients, alleviating edema induced by inflammation to maintain the depth of the sulcus depth. This might be relevant to the maintenance of normal cognitive function, but whether it involves compensatory mechanisms needs to be explored and proved further.

The gyrification index and fractal dimension are commonly utilized as key indices of cortical complexity, and aberrations in these parameters may serve as biological markers for neuropsychiatric disorders (King et al., 2010; Matsuda and Ohi, 2018), but the conclusions are controversial. Recently research showed individuals with T2DM had higher gyrification index than healthy participants (Crisóstomo et al., 2021), while another study revealed that the gyrification index is lower in T2DM patients with mild cognitive impairment (Shao et al., 2022). Some experts suggest that the relationship between gyrification index and cognitive function is changing dynamically during the progression of neurodegenerative diseases, and also that alterations in various brain areas are not synchronized (Lebed et al., 2012; Núñez et al., 2020). Fractal dimension is a morphological variability sensitive index used to evaluate brain structural complexity (Chen et al., 2022). Decreased fractal dimension has been found in mild cognitive impairment patients and AD patients (Ruiz de Miras et al., 2017; Nicastro et al., 2020). On the alterations in fractal dimension in T2DM patients, however, very few studies have been conducted. We speculate that the fractal dimension, like the gyrification index, undergoes dynamic changes during the progression of cognitive function alterations in T2DM patients.

Inevitably, there are some limitations to our study. First off, our sample size is somewhat limited because of the stringent inclusion and exclusion criteria. Based on this research, we would continue to increase the sample size in future investigations. Secondly, our study was a cross-sectional study, we intend to conduct long-term follow-up observations on the subjects in the future to capture the key nodes of cognitive impairment in T2DM patients. Thirdly, due to the complicated medication regimen for patients with T2DM, we could not take the patients’ medication situation into consideration in this study. How to evaluate the influence of medicine use in future studies related to T2DM is a topic worth exploring.

## 5. Conclusion

T2DM patients without cognitive impairment displayed reductions in IL 4 and BDNF levels, as well as significant alterations in their SBM indices, indicating that prior to the emergence of cognitive impairment, the SBM indices, peripheral cytokines, and BDNF may have altered in T2DM patients. IL-10 may lessen inflammation-related brain edema and preserve sulcus depth in T2DM patients through its anti-inflammatory activity. The combination of inflammatory biomarkers and MRI may yield valuable perspectives on comprehending the mechanisms of cognitive impairment in T2DM individuals. Further study with expanded sample size and follow-up investigations should be carried out to establish if cytokines, BDNF, and the SBM indices are involved in the process of compensating for cognitive function ahead of the onset of T2DM-related cognitive impairment.

## Data availability statement

The raw data supporting the conclusions of this article will be made available by the authors, without undue reservation.

## Ethics statement

The studies involving human participants were reviewed and approved by Medical Research Ethics Committee of Guangzhou University of Chinese Medicine (No. K2019-143). The patients/participants provided their written informed consent to participate in this study. Written informed consent was obtained from the individual(s) for the publication of any potentially identifiable images or data included in this article.

## Author contributions

WL: data curation, methodology, formal analysis, investigation, writing – original draft, and visualization. YC and KZ: data curation and investigation. XT: conceptualization and validation. YW: methodology, validation, visualization, and writing – review and editing. SQ: conceptualization, funding acquisition, project administration, resources, supervision, and writing – review and editing. All authors contributed to the article and approved the submitted version.

## Footnotes

1
http://www.fil.ion.ucl.ac.uk/spm

2
http://www.neuro.uni-jena.de/cat/

3
https://jasp-stats.org/

4
https://www.rstudio.com/
